# A Handling Study to Assess Use of the Respimat^®^ Soft Mist™ Inhaler in Children Under 5 Years Old

**DOI:** 10.1089/jamp.2014.1159

**Published:** 2015-10-01

**Authors:** Wolfgang Kamin, Marion Frank, Sabine Kattenbeck, Petra Moroni-Zentgraf, Herbert Wachtel, Stefan Zielen

**Affiliations:** ^1^ Children's Hospital, Evangelisches Krankenhaus Hamm, Hamm, Germany.; ^2^ Boehringer Ingelheim, Ingelheim, Germany.; ^3^ Children's Hospital, Allergology, Pneumology and Cystic Fibrosis, Goethe-University, Frankfurt am Main, Germany.

**Keywords:** device handling, inhalation flow profiles, preschool children, Respimat^®^ Soft Mist™ Inhaler, respiratory disease

## Abstract

***Background:*** Respimat^®^ Soft Mist™ Inhaler (SMI) is a hand-held device that generates an aerosol with a high, fine-particle fraction, enabling efficient lung deposition. The study objective was to assess inhalation success among children using Respimat SMI, and the requirement for assistance by the parent/caregiver and/or a valved holding chamber (VHC).

***Methods:*** This open-label study enrolled patients aged <5 years with respiratory disease and history of coughing and/or recurrent wheezing. Patients inhaled from the Respimat SMI (air only; no aerosol) using a stepwise configuration: “1” (dose released by child); “2” (dose released by parent/caregiver), and “3” (Respimat SMI with VHC, facemask, and parent/caregiver help). Co-primary endpoints included the ability to perform successful inhalation as assessed by the investigators using a standardized handling questionnaire and evaluation of the reasons for success. Inhalation profile in the successful handling configuration was verified with a pneumotachograph. Patient satisfaction and preferences were investigated in a questionnaire.

***Results:*** Of the children aged 4 to <5 years (*n*=27) and 3 to <4 years (*n*=30), 55.6% and 30.0%, respectively, achieved success without a VHC or help; with assistance, another 29.6% and 10.0%, respectively, achieved success, and the remaining children were successful with VHC. All children aged 2 to <3 years (*n*=20) achieved success with the Respimat SMI and VHC. Of those aged <2 years (*n*=22), 95.5% had successful handling of the Respimat SMI with VHC and parent/caregiver help. Inhalation flow profiles generally confirmed the outcome of the handling assessment by the investigators. Most parent/caregiver and/or child respondents were satisfied with operation, instructions for use, handling, and ease of holding the Respimat SMI with or without a VHC.

***Conclusions:*** The Respimat SMI is suitable for children aged <5 years; however, children aged <5 years are advised to add a VHC to complement its use.

## Introduction

Inhalers are essential for noninvasive delivery of drugs directly to the lungs, and a range of devices are available. Successful inhalation therapy depends on a number of different parameters, including the age and health status of the patients, the dose of the drug, and its distribution in the lung.^(1–3)^ Correct handling of the specific inhaler is required for patients to achieve an adequate dose.^(3,4)^ Studies show that most devices are effective if used correctly;^(1)^ however, incorrect use of inhalers is common and reduces the clinical efficacy of inhaled medications in many patients.^(3,5,6)^

In young children, cooperation with parents/caregivers and the ability to perform an inhalation maneuver correctly are critical to successful inhalation therapy.^(3,7)^ Lack of coordination of actuation and inhalation is a common problem in children using pressurized metered-dose inhalers (pMDIs), with inhalation being either too early or too late.^(3)^ Indeed, many studies show that improper inhaler technique is common in children, resulting in reduced or no benefit.^(6,8,9)^ In addition, young children may be unable to generate sufficient airflow required by some devices.^(3,5)^

The ideal inhaler for young children would be an active device that is easy to handle, with no requirement for the coordination of actuation and inhalation, an emitted dose delivered effectively to the lungs independent of inspiratory flow, and a formulation suitable for use with a valved holding chamber with facemask (VHC).^(3)^ The Respimat^®^ Soft Mist™ Inhaler (SMI) is a novel, handheld, propellant-free, multidose inhalation device that generates a slow-moving, long-lasting aerosol plume containing a large fine-particle fraction (FPF <5 μm) that enables efficient drug delivery to the lungs.^(10–12)^ The emitted dose is independent of the inspiratory flow and the Respimat SMI is easy to use.

In studies of adults, the Respimat SMI has been well accepted, mainly due to its inhalation and handling characteristics, and satisfaction with the Respimat SMI is greater than with pMDIs or dry powder inhalers (DPIs).^(13,14)^ Studies have also shown that most children aged >5 years are able to perform correct inhalation with Respimat SMI;^(10,15)^ however, no previous studies report its suitability in the treatment of children aged <5 years. The aim of the current study was to evaluate the use of the Respimat SMI in children aged <5 years.

## Materials and Methods

### Study design

This was a two-center, open-label, noninvasive handling study with a sequential design that was conducted in the Universities of Mainz and Frankfurt, Germany, between October 2008 and April 2009. The objective of the study was to assess the success of inhalation maneuvers among children aged <5 years using a Respimat SMI, and the degree to which assistance by the parent/caregiver and/or a VHC is appropriate for performing a successful inhalation maneuver. The study was conducted in accordance with Good Clinical Practice, applicable regulatory requirements, and the principles set forth in the Declaration of Helsinki (1996). This was not a clinical trial according to the German Drug or Medical Devices Act, as no medication was administered, but institutional review board approval was obtained.

### Study participants

The study enrolled patients aged <5 years, of either sex, with any respiratory disease and a history of coughing and/or recurrent wheezing; patients had to be able to perform a technically acceptable inhalation with the pneumotachograph. Parents of all patients had to provide informed consent consistent with International Conference on Harmonisation-Good Clinical Practice guidelines prior to the child's participation. Patients were excluded if they had significant diseases other than pulmonary diseases, or if they had tuberculosis, acute illness and fever above 38.5°C, or oxygen saturation <94%.

### Device and training

Training on the handling of the Respimat^®^ SMI (Boehringer Ingelheim Pharma GmbH & Co KG, Ingelheim, Germany) was given to the children and their parents/caregivers by an appropriately trained member of the site staff before first use, and included written instructions, oral instructions, display of handling video, and a handling demonstration with the Respimat SMI.

The training was standardized and provided by highly motivated staff members between the ages of 22 and 28 years. The Respimat SMI user was required to breathe out slowly and deeply, close his/her lips around the end of the mouthpiece (while holding the inhaler horizontally), and press the dose-release button while taking a single slow, deep breath in through the mouth. When used with a VHC (AeroChamber Plus^®^ with facemask, Trudell Medical International, London, Ontario, Canada), tidal breathing was permitted (with a target of five breaths). In this study, no aerosol was generated and patients inhaled air when conducting inhalation maneuvers.

**FIG. 1.**
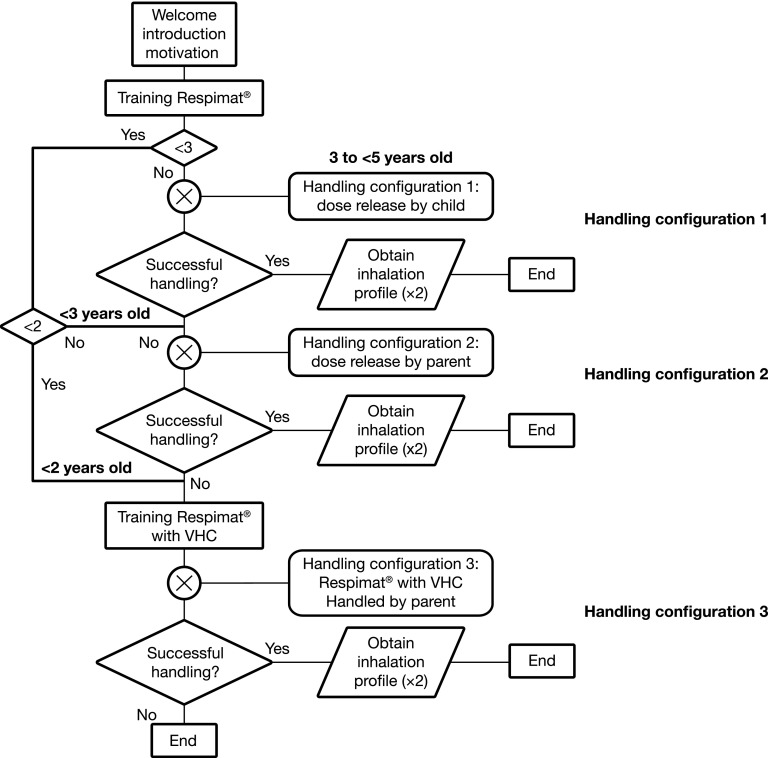
Flow chart of handling test procedure. Children aged ≥3 to <5 years started with handling configuration 1 (Respimat SMI alone). Children aged 2 to <3 years started with handling configuration 2 (Respimat SMI with parent/caregiver help). Children aged <2 years started with handling configuration 3 (Respimat SMI with valved holding chamber, facemask, and parent/caregiver help). The child was allowed a maximum of three attempts to use the inhaler in each configuration. If patients aged ≥2 years were unsuccessful in using a handling configuration, they attempted the next configuration.

Children were divided into four age groups (0 to <2 years, 2 to <3 years, 3 to <4 years, and 4 to <5 years) to assign suitable handling configurations that would avoid the need for all children to perform a high number of inhalation maneuvers using a stepwise approach (Fig. 1). Children aged ≥3 years started with handling configuration 1, in which the Respimat SMI was used without a VHC and without help by the parent/caregiver. Children aged 2 to <3 years started with handling configuration 2, in which the Respimat SMI was used without a VHC but with help by the parent/caregiver. Children aged <2 years started with handling configuration 3, in which the Respimat SMI was used with a VHC and with help by the parent/caregiver. After receiving an explanation of the device, the child was allowed a maximum of three attempts to use the inhaler in each configuration. If patients aged ≥2 years were unsuccessful in using a handling configuration, they attempted the next configuration.

Handling was assessed by trained personnel using a standardized questionnaire, with successful handling defined as enclosure of the inhaler mouthpiece without covering the air vents; coordination of dose release and inhalation; and, when used with VHC, correct placement of the VHC with facemask on the child's mouth and nose, followed by inhalation. Once a successful handling configuration was identified, it was repeated with a dedicated test set-up permitting the recording of inhalation flow profiles. The criterion for a successful inhalation flow profile was a minimum volume inhaled (V_in_) of 0.15 L.

### Assessments

The co-primary endpoints included the proportion of patients able to perform successful inhalation maneuvers, as evaluated by the investigators using a standardized handling questionnaire, and assessment of the scores from this questionnaire was used to obtain some information on the reason for unsuccessful handling.

**FIG. 2.**
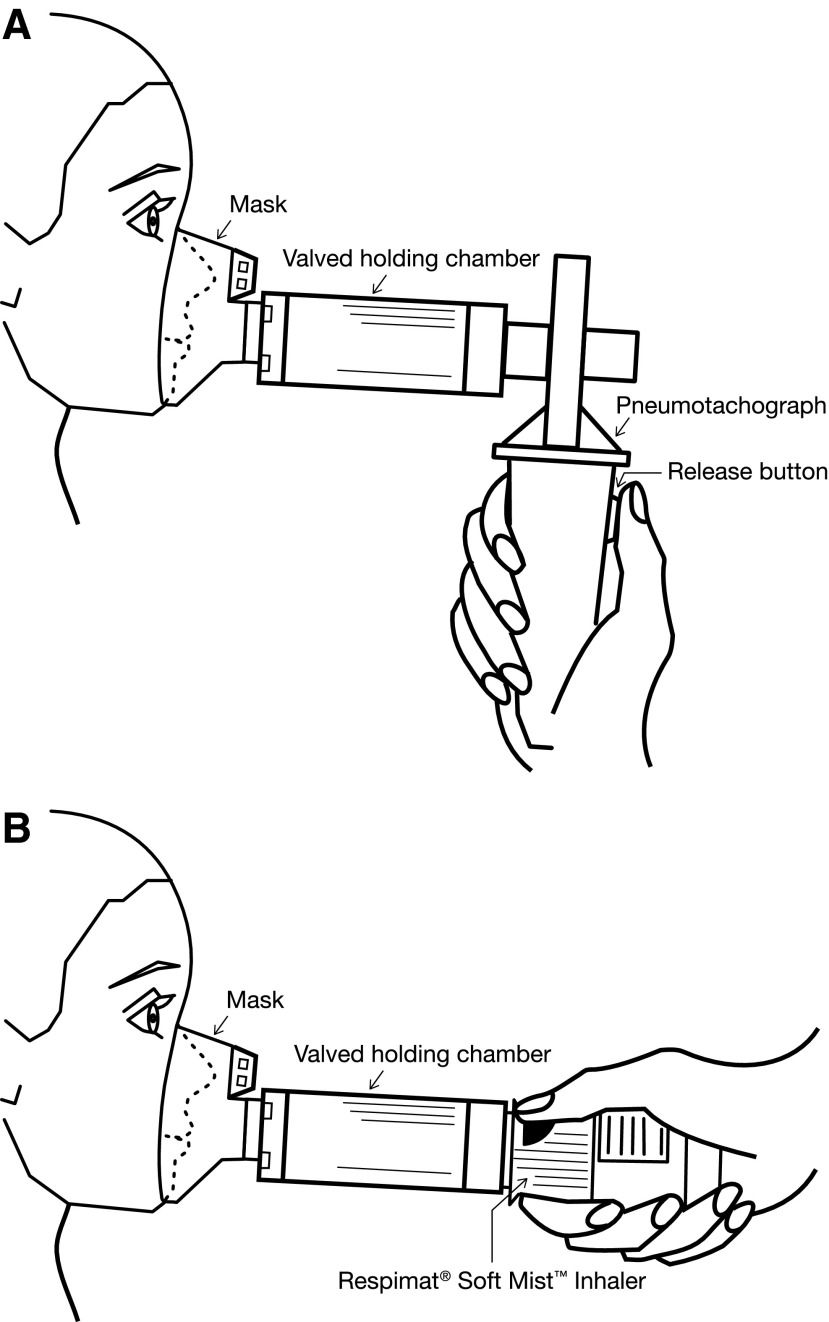
**(A)** Valved holding chamber (VHC) coupled to the pneumotachograph handle. The connector simulated the flow resistance of the Respimat SMI air vents and the handle was equipped with a release button which had to be pressed for simulating the release of a dose. (The Respimat SMI was not included in this setting). **(B)** Use of VHC with Respimat.

Secondary endpoints included inhalation flow profile parameters and inhalation flow profiles as an alternative to assess correct inhalation. The inhalation profile in the successful handling configuration was verified with a pneumotachograph (JAEGER^®^ MasterScope, CareFusion Corporation, Hoechberg, Germany) for the successful handling configurations (Fig. 2). The volume of the VHC (0.15 L) when emptied by the target number of breaths (∼5) was used as a criterion for inhalation success. For consistency and in accordance with the young age of the patients, the acceptance threshold for the V_in_ after pushing the dose-release button during specified time periods (1.5 seconds, corresponding to spray duration for handling configurations 1 and 2, and a decay half-time for the aerosol cloud of 10 seconds when using the VHC in handling configuration 3)^(16)^ was therefore set to ≥0.15 L. Another secondary endpoint was the evaluation of patient satisfaction and preferences using a separate questionnaire (comprising 10 questions) completed by the parent/caregiver and children together.

### Statistical analyses

Descriptive statistics were applied and all data were analyzed using SAS (SAS Institute Inc., Cary, NC, USA). No replacement of missing data was made. Because of the exploratory nature of the study, there was no formal statistical sample size calculation. However, a total of 80 patients (20 for each of the four age groups) was considered adequate to obtain sufficient data.

## Results

A total of 103 children were enrolled, with 99 entering and completing the study (0 to <2 years, *n*=22; 2 to <3 years, *n*=20; 3 to <4 years, *n*=30; 4 to <5 years, *n*=27). Four children were excluded because they did not adhere to study protocol (they did not pay sufficient attention during the instruction process). Baseline demographics, status of current diseases, and details of previous use of inhalation devices are shown in Table 1. Most of the children were male (63%) and Caucasian (87%). They had various obstructive lung diseases, including asthma, bronchitis, and cystic fibrosis (Table 1). A majority (89%) of the 103 children enrolled had previous experience with inhalation devices: nebulizer, 14%; nebulizer plus mask, 44%; pMDI, 6%; pMDI plus VHC, 8%; pMDI plus VHC and mask, 17%.

**Table 1.** Demographic Data, Current Diseases, and Previous Inhalation Devices Used at Baseline

	*Age group (years)*				
	*All*	*0 to <2*	*2 to <3*	*3 to <4*	*4 to <5*
Number of patients	103	25	20	30	28
Males	65 (63)	18 (72)	11 (55)	17 (57)	19 (68)
Race					
Caucasian (European)	90 (87)	23 (92)	16 (80)	24 (80)	27 (96)
Caucasian (non-European)	1 (1)	1 (4)	0 (0)	0 (0)	0 (0)
Asian	9 (9)	0 (0)	2 (10)	6 (20)	1 (4)
Black	3 (3)	1 (4)	2 (10)	0 (0)	0 (0)
Diagnosis					
Preschool asthma	67 (65)	17 (68)	12 (60)	17 (57)	21 (75)
Recurrent bronchitis	26 (25)	6 (24)	5 (25)	12 (40)	3 (11)
Cystic fibrosis	10 (10)	2 (8)	3 (15)	1 (3)	4 (14)
Device^*^	197	44 (100)	34 (100)	66 (100)	53 (100)
No device	–	9 (21)	3 (9)	14 (21)	16 (13)
Nebulizer	–	–	8 (24)	9 (14)	9 (17)
Nebulizer+mask	–	31 (71)	18 (53)	21 (32)	11 (21)
pMDI	–	–	–	2 (3)	5 (9)
pMDI+VHC	–	–	1 (3)	5 (8)	5 (9)
pMDI+VHC+mask	–	4 (9)	4 (12)	15 (23)	7 (13)

All data are *n* (%).The safety data set as well as the full analysis data set consisted of all (*N*=103) patients. Four patients had protocol violations and so were excluded from the per protocol data set (*N*=99). ^*^Results are inclusive of patients who may have used more than one device.

pMDI, pressurized metered dose inhaler.

### Outcomes (and scores) of handling questionnaire assessments

Children aged 4 to <5 years started with configuration 1. Fifteen of the 27 children (56%) in this age group achieved success using the Respimat SMI without the VHC or parent's/caregiver's help. With assistance from parents/caregivers (configuration 2), another eight children (30%) could handle the Respimat SMI without the VHC. With the VHC added, the remaining four (15%) 4 to <5 year olds all achieved success (configuration 3) (Table 2). Children aged 3 to <4 years also started with handling configuration 1. Nine of the 30 children (30%) achieved success using the Respimat SMI without the VHC and without help from the parent/caregiver. After transfer to configuration 2 (i.e., adding assistance from parents/caregivers), another three (10%) could handle the Respimat SMI without the VHC. The remaining 18 (60.0%) children aged 3 to <4 years all achieved a successful handling maneuver when transferred to configuration 3 (i.e., with VHC added) (Table 2). Children aged 2 to <3 years started with configuration 2 (Respimat SMI without VHC but with parent's/caregiver's help), but none of the 20 could correctly use the Respimat SMI without the VHC. All were successful in handling configuration 3 (with both VHC and parent's/caregiver's help) (Table 2). Children aged <2 years started with handling configuration 3 (Respimat SMI with both VHC and parent's/caregiver's help), and only one of the 22 children was unsuccessful due to noncooperation (Table 2).

**Table 2.** Proportion (% [n/N]) of Patients Able to Perform Successful Handling as Assessed by the Standardized Handling Questionnaire Followed by Assessment of Inhalation Flow Profiles

	*Handling questionnaire*			*Inhalation flow profiles*	
*Handling configuration*^a^	*1*	*2*	*3*	*1 and 2*	*3*
Age 0 to <2 years	–	–	96 (21/22)	–	67 (14/21)
Age 2 to <3 years	–	–	100 (20/20)	–	90 (18/20)
Age 3 to <4 years	30 (9/30)	10 (3/30)	60 (18/30)	55 (6/11)^b^	100 (19/19)^b^
Age 4 to <5 years	56 (15/27)	30 (8/27)	15 (4/27)	83 (19/23)^c^	100 (5/5)^c^

^a^Configurations: 1 (Respimat SMI alone); 2 (Respimat SMI with parent/caregiver help); 3 (Respimat SMI with VHC, facemask, and parent/caregiver help); ^b^One patient passed configurations 1 and 2 and moved to configuration 3 during the inhalation flow profiles; ^c^One patient delivered inhalation profiles with and without VHC (i.e., *n*=28).

Analysis of the scores of the handling questionnaire revealed that in children aged 4 to <5 years, lack of success in configuration 1 was due to unsuccessful enclosing of the mouthpiece (17%), unsuccessful release (75%), or unsuccessful inhalation (8%). In children aged 3 to <4 years, lack of success in configuration 1 (Respimat SMI alone) was due to unsuccessful enclosing (62%), unsuccessful release (29%), or unsuccessful inhalation (10%). In children aged ≥3 years, if assistance from the parent/caregiver was provided, coordination of dose release with inhalation was improved. Unsuccessful handling of the Respimat SMI by children aged 2 to <3 years in configuration 2 (Respimat SMI alone with parent's/caregiver's help) was due to unsuccessful enclosing (55%), unsuccessful release (no button press or accidental release, 10%), or difficulties with coordinating dose release with inhalation (35%).

### Inhalation flow profiles

Inhalation flow profiles were recorded in all age groups, and the parameters measured during the handling configurations are outlined in Figure 2A (configurations 1 and 2) and Figure 2B (configuration 3). In handling configurations 1 and 2 (Table 3), median V_in_ was 0.63 L in children aged 3 to <4 years and 0.47 L in those aged 4 to <5 years. Median values for difference between time of actuation and start of inspiration (Delta) were between −0.2 and −0.3 seconds in handling configurations 1 and 2, and median total inhalation time (T_in_) was between 1.0 and 1.2 seconds. Median values for time between dose release and subsequent breath (Delta2) were between −0.6 and −2.6 seconds for handling configuration 3. The median peak inspiratory flow (PIF) reached 1 L/sec. In handling configuration 3 (Table 4), Respimat SMI with use of the VHC, median values of V_in_ ranged from 0.34 L to 1.12 L across the age groups. In handling configuration 3, PIF did not surpass 1 L/s.

**Table 3.** Inhalation Profile Parameters With Handling Configurations 1 or 2 (the Respimat SMI Without or With Parent/Caregiver Assistance in Children Aged ≥3 Years)

	*Age group (years)*							
	*3 to <4*				*4 to <5*			
	n	*Median*	*Min*	*Max*	n	*Median*	*Min*	*Max*
Delta^a^ (sec)	6	−0.210	−0.452	0.308	19	−0.299	−1.023	0.790
T_in_^b^ (sec)	6	1.162	0.935	1.295	19	0.979	0.502	2.373
VC_in_^c^ (L)	6	0.681	0.437	0.960	19	0.637	0.324	1.153
V_in_^d^ (L)	6	0.633	0.388	0.903	19	0.465	0.255	1.022
PIF^e^ (L/sec)	6	1.015	0.600	1.900	19	1.000	0.540	2.140

^a^Time span between pressing the dose-release button and start of inhalation, with negative Delta indicating early release; ^b^Total duration of inhalation; ^c^Total inhaled volume; ^d^Volume inhaled during 1.5 sec after the release of the dose; ^e^Peak inspiratory flow rate observed in the flow profiles during inhalation.

**Table 4.** Inhalation Profile Parameters With Handling Configuration 3 (the Respimat^®^ SMI With VHC, With Parent/Caregiver Assistance)

	*Age group (year)*															
	*0 to <2*				*2 to <3*				*3 to <4*				*4 to <5*			
	N	*Median*	*Min*	*Max*	N	*Median*	*Min*	*Max*	N	*Median*	*Min*	*Max*	N	*Median*	*Min*	*Max*
Delta^a^ (sec)	14	−1.788	−7.39	2.041	18	−0.283	−6.45	6.115	19	0.164	−4.13	1.494	5	−0.371	−4.40	3.593
Delta2^b^ (sec)	14	−2.611	−7.39	−0.124	18	−1.025	−6.45	−0.088	19	−1.528	−4.13	−0.096	5	−0.593	−4.40	−0.371
Count^c^ (−)	14	5	2	7	18	5	1	10	19	4	2	7	5	4	3	8
T_pulse^d^ (sec)	14	0.263	0.141	0.495	18	0.345	0.261	1.162	19	0.437	0.183	0.901	5	0.416	0.409	0.809
f^e^ (Hz)	14	0.742	0.341	1.167	18	0.546	0.202	1.255	19	0.448	0.330	0.681	5	0.511	0.355	0.729
VC_in_^f^ (L)	14	0.570	0.253	1.155	18	1.031	0.548	2.931	19	1.445	0.271	2.712	5	1.724	0.871	3.285
V_in_^g^ (L)	14	0.342	0.188	0.776	18	0.711	0.300	1.713	19	1.121	0.187	2.120	5	0.940	0.409	2.415
PIF^h^ (L/sec)	14	0.335	0.150	1.030	18	0.445	0.200	0.990	19	0.470	0.140	0.880	5	0.690	0.190	0.850

^a^Time span between pressing the dose-release button and start of inhalation, with negative Delta indicating early release; ^b^Time between a dose release and subsequent breath; ^c^Number of inhalation breaths recorded within 10 sec after release or until the end of recording, whichever occurred earlier; ^d^Average inhalation time calculated from the multiple breaths during use of the VHC.

^e^Frequency (Hz) of inhalation breaths as determined by fast Fourier transform of the inhalation profile. It is the inverse of the average time between repetitive breaths; ^f^Volume accumulated during breathing several breaths (target five); ^g^Volume inhaled during 10 seconds after the release of the dose; ^h^Maximum peak inspiratory flow observed in the flow profiles during inhalation after pressing the dose-release button.

**FIG. 3.**
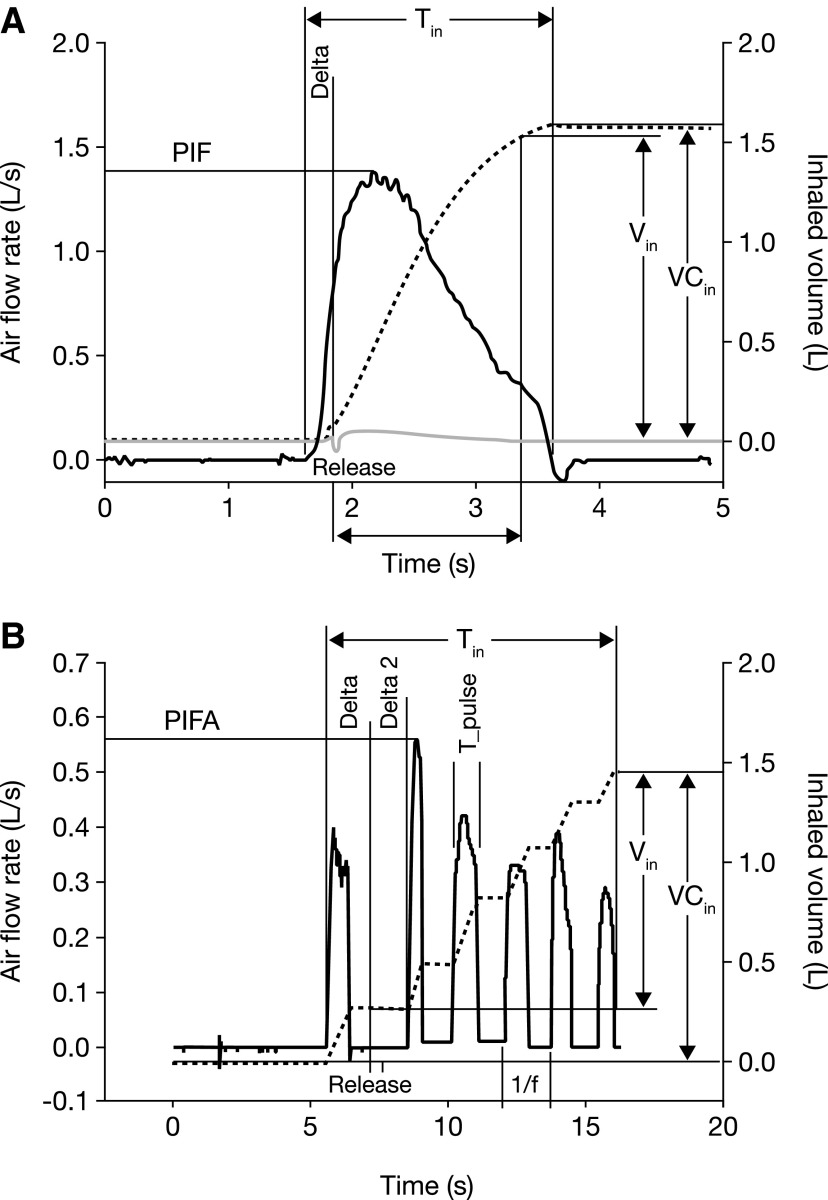
Schematic representation of parameters acquired from inhalation flow profiles during **(A)** handling configurations 1 and 2 or **(B)** handling configuration 3. PIF, peak inspiratory flow; PIFA, peak inspiratory flow after release.

The inhalation flow profiles in patients with successful outcomes (based on the handling questionnaire) are summarized in Table 2, and shown diagrammatically in Figure 3. For patients aged 0 to <2 years, 67% of those who could use Respimat SMI with VHC (configuration 3) achieved a successful inhalation flow profile. Of patients aged 2 to <3 years, 90% who could use Respimat SMI with VHC achieved successful inhalation flow profiles. For those aged 3 to <4 years, 40% could use Respimat SMI without VHC (configurations 1 and 2), and 55% of these achieved adequate inhalation. For those aged 4 to <5 years, 85% could use Respimat SMI without VHC, and 83% of these had adequate inhalation. All patients aged >3 years who could use Respimat SMI with VHC achieved adequate inhalation.

Reasons for unsuccessful handling in children <3 years of age included difficulties with enclosing the mouthpiece of the Respimat^®^ SMI correctly and also with coordination of dose release with inhalation, even with help from parents/caregivers.

Enclosing the mouthpiece (3 to <4 years, unsuccessful 62%) and coordination of dose release with inhalation (4 to <5 years, unsuccessful 75%) were difficult steps among older children performing the handling without help. In general, inhalation worked well in these age groups (successful inhalation: 3 to <4 years, 90%; 4 to <5 years, 92%). Children in the age groups (3 to <5 years) who did not pass the handling test without help of parents/caregivers made the most mistakes in handling steps 3 (53%) and 5 (35%)(i.e., enclosing the mouthpiece and inhaling).

### Outcomes of satisfaction and preferences questionnaire

**FIG. 4.**
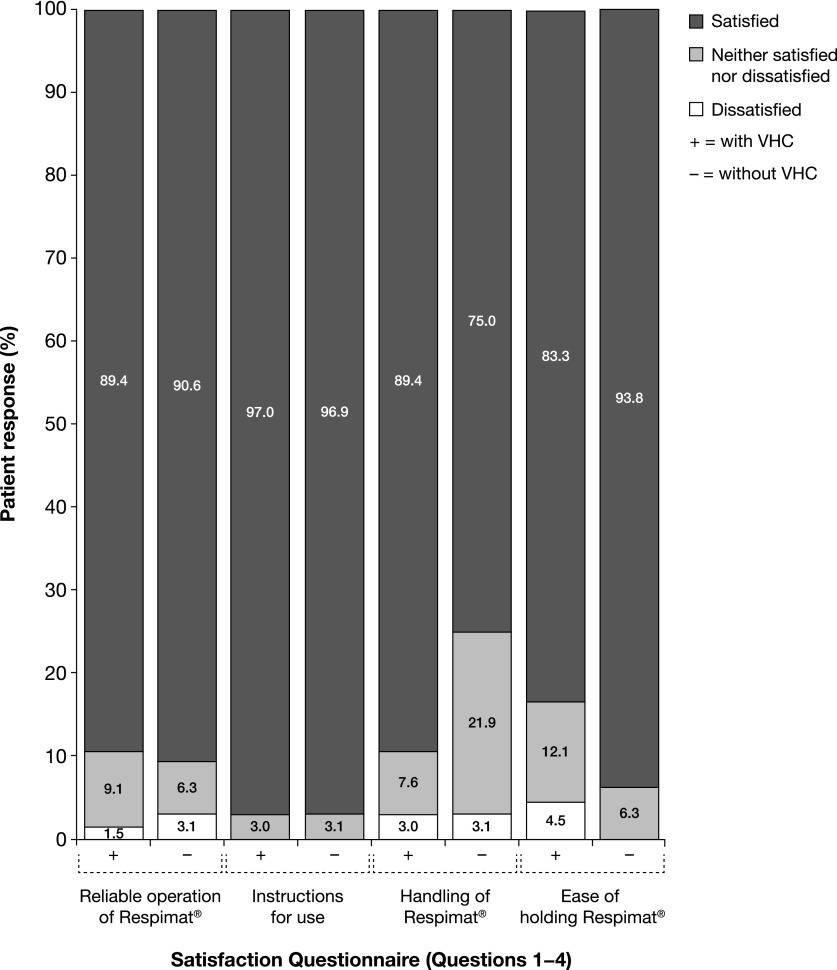
Parent's/caregiver's responses to satisfaction components of the satisfaction and preferences questionnaire. Questions were rated with a seven-point Likert scale. For easier interpretation of the results the grades were grouped as follows: satisfied (grades 1 and 2: very satisfied and satisfied); neither satisfied nor dissatisfied (grades 3, 4, and 5: somewhat satisfied, neither satisfied nor dissatisfied, somewhat dissatisfied); and dissatisfied (grades 6 and 7: dissatisfied and very dissatisfied).

After the handling test, most of the participants, together with their parent/caregiver, completed a questionnaire concerning satisfaction and preferences. Analysis of the responses showed that, regardless of the configuration (i.e., handling with or without VHC), the vast majority of children and parents/caregivers were satisfied with operation, instructions for use, handling, and ease of holding the Respimat SMI (Fig. 4). Furthermore, approximately 90% of participants were satisfied with the reliable operation of the Respimat SMI, and >95% declared satisfaction with instructions for its use. Without the VHC, 75% of participants reported satisfaction with handling of Respimat SMI, and >90% were satisfied with ease of holding during use. With or without the VHC, >90% of parents/caregivers felt that they and their child would be able to manage the handling of the Respimat SMI; almost half stated that the handling of the Respimat SMI was better than that of their current device.

When asked “What do you like best?” parents'/caregivers' replies were mainly the quick and simple handling of the Respimat SMI (40% and 21% of answers, respectively), its small size (13%), and dose indicator (2%). When asked “What do you dislike?” parents'/caregivers' replies were mainly coordination problems (14% of answers) and difficulties in handling for children (7%). Around 50% of answers from parents/caregivers indicated that they had no criticism.

A small number of older children (*n*=27, 4 <age group <5) were able to provide answers to these questions, and 10 children stated that they liked turning the clear base of the Respimat SMI the most, four liked the click during release, and two liked the color and design most. The question for criticism received eight answers. They were limited mainly to difficulty opening the cap (two answers), fragility, and too small arrows (one answer each); four of the children who answered this question indicated no criticism.

## Discussion

Successful inhalation therapy in young children depends on cooperation, the technical properties of the inhalation device, and the ability of the child to perform a correct inhalation maneuver with the device.^(3,7,11)^ Several features of the Respimat SMI recommend its use specifically in the pediatric population. Respimat SMI is an active inhaler, making supervised inhalation relatively easy, and resulting in an emitted dose that is independent of the inspiratory flow. Dose inhalation is much faster with the Respimat SMI (seconds) compared with nebulizers (∼10 min), and the slow aerosol delivery with Respimat minimizes hand–breath coordination requirements compared with pMDIs. Importantly, the Respimat SMI is suitable for use with a VHC by young children, to ease inhalation.

Ease of Respimat SMI use has been demonstrated previously in adults and in children aged >5 years.^(13–15,17)^ In this study, for the first time we assessed Respimat SMI handling in children aged <5 years. The main finding of the study was that preschool children are able to inhale from the Respimat SMI successfully. More than half of the children aged 4 to <5 years could use it without a VHC or help, and, although a minority of children aged <5 years experienced problems with VHC and without the help of a parent/caregiver, all children achieved success with assistance from the parent/caregiver and the use of a VHC. These results suggest that the Respimat SMI is suitable for use in young children when given help appropriate to their age.

As indicated in the Introduction, successful inhalation without and, to a limited extent, with VHC is reported in the literature as being difficult, mainly because of insufficient coordination and/or leakage induced (e.g., by mask rejection). The extraordinarily high success rates obtained with Respimat are attributed by the authors to the following features: age-appropriate study design with age-related configurations, well-trained and enthusiastic study personnel, standardized training of the study participants and their caregivers, and success-check based on objective flow profiles. Instruction from young, passionate assistants, who followed a standardized training program, was perceived as particularly key in the successful device tuition evidenced in this study, as the children responded very well to such personal contact and level of individual care.

Regarding inhalation success, the flow profiles generally confirmed the handling assessment with the standardized questionnaire across all age groups and configurations. This indicates that sufficient drug may be inhaled with successful handling. The inhalation flow profiles showed that the median time difference between pressing the dose-release button and the start of inhalation (Delta)/subsequent breath (Delta2) was between −0.3 and −2.6 sec. This was considered acceptable since a reasonable amount of the spray stays in the VHC for a half-time of approximately 10 sec (specific value for the AeroChamber^®^).^(16)^ V_in_ with a VHC was greater than without, as approximately five breaths are required with the VHC. Without a VHC, only one single inhalation maneuver takes place in order to administer the output of one release. This could, in part, explain why the median V_in_ was higher in 3 to <4 year olds compared with 4 to <5 year olds; however, a difference in the number of patients included in the two age groups (6 versus 19, respectively) could account for this, as it would be expected that older patients would have had greater V_in_.

In contrast to many other devices, the Respimat SMI has the potential to facilitate coordination of actuation with inhalation, as the spray duration is relatively long (1.5 sec). The T_in_ in children using handling configurations 1 and 2 was approximately 1 sec (and therefore shorter than the spray duration of Respimat SMI). As there is a linear correlation between lung deposition in the upper airways and flow velocity,^(17)^ mean flow (as indicated by total inhaled volume [VC_in_]/T_in_) is more of a key parameter of inhalation success. PIF is less relevant to the Respimat SMI than to other inhalers such as DPIs, as the Respimat SMI is not dependent on PIF for aerosol generation.^(15,17)^ Nevertheless, to optimize drug delivery, training in the use of the Respimat SMI should focus on exhalation followed by slow, long-lasting, and deep inhalation.

These findings in children aged <5 years are consistent with previous studies of the Respimat SMI in older pediatric populations.^(10,15)^ The first pediatric handling study was in 99 children aged 4–12 years, and showed that the use of the Respimat SMI without the VHC and without help from parents was suitable for children aged ≥5 years.^(15)^ Based on air-flow profile recordings (drug was not given), 70% of children aged 4–8 years and 83% of children aged 9–12 years successfully handled the Respimat SMI without parental help. Four-year-old children did not reach the minimum air volume required in the study, but the effect of adding parental help was not assessed. A clinical study in pediatric patients showed that most children aged >5 years were able to perform correct inhalation of an active substance with the Respimat SMI.^(10)^ In this multicenter, randomized, double-blind study, 535 children aged 6–15 years with asthma were treated with ipratropium/fenoterol in fixed-dose combination using either Respimat SMI without VHC or chlorofluorocarbon MDI with VHC to take the study medication three times daily for 4 weeks. There were no issues regarding ability to use the Respimat SMI or accidental exposure. The Respimat SMI was at least as effective as a chlorofluorocarbon MDI with VHC and safety in the two groups was comparable.^(10)^ Previous controlled clinical studies of the Respimat SMI in adults with chronic obstructive pulmonary disease (COPD) or asthma have supported its ease of use, with good retention of technique following training and a high degree of patient satisfaction.^(12,14,18)^

Analysis of satisfaction and preferences in this study showed that most children and their parents/caregivers were satisfied with operation, instructions for use, handling, and ease of holding of the Respimat SMI, regardless of the handling configuration. In particular, parents/caregivers favored the Respimat SMI because of its quick and simple handling, and they also liked its small size and dose indicator. These findings are in agreement with satisfaction data obtained for the Respimat SMI in adult patients.^(13)^ Some studies compared the Respimat SMI with other inhalers in patients with COPD^(13)^ and found that the Respimat SMI produced significantly higher scores for patient total satisfaction compared with pMDIs, and also significantly higher total satisfaction scores versus a DPI. Another study showed that most children prefer the Respimat SMI over hydrofluoroalkane (HFA)-MDI following inhaler technique training.^(18)^ Data showed that 81% of children preferred the Respimat SMI over HFA-MDI, and a significantly higher proportion of children would rather continue the Respimat SMI (*p* <0.001). Satisfaction scores (both total and 13 or 15 domains) were significantly higher with the Respimat SMI than with HFA-MDI (*p* <0.05).

The design of this study had several limitations. First, the study generated qualitative, not quantitative, results with respect to identification of critical handling steps. A child who did not pass a certain critical handling step was not tested in subsequent steps in the same handling configuration, and instead moved to the next configuration. This leads to an apparent over-reporting of failures in the early handling steps. Second, it was not possible to gain any information on possible learning effects because assessment took place on one day only. In practice, children who use a certain inhaler over the long-term or receive repeated training might perform better. Despite these limitations, this is the first study that investigated, and has demonstrated, that the Respimat SMI is suitable for use in children aged <5 years. The inhalation-flow profiles obtained in this study will be used in combination with future *in vitro* studies to enable the calculation of the theoretical dose to the lung for this age group. In addition, data from handling studies with flow profile assessments, together with pharmacokinetic data, can support and complement clinical efficacy and safety data and assist inhaler development. This approach is particularly useful in young children, as obtaining scintigraphic data is difficult to justify ethically in this population.

In conclusion, the Respimat SMI is a new-generation inhaler suitable for patients with chronic respiratory diseases, with design and operational features that are desirable for use in the pediatric population. This handling study demonstrated that the Respimat SMI is suitable for inhalation therapy in young children (aged <5 years). Results from the standardized questionnaire performed by the investigators and assessment of flow profiles suggested that children aged <4 years should use the Respimat SMI with a VHC. In addition, while the majority of 4- to <5-year-old patients could handle the Respimat SMI without a VHC, there was considerable variability in the inhalation flow-profile parameters; thus, to ensure standardized dosing, use of the Respimat SMI with a VHC is recommended for all children aged <5 years. This trial also supports the use of Respimat SMI (with and without VHC) in clinical trials of inhaled drug therapy in young children.
